# The Digestibility of Vegan and Vegetarian Diets for Dogs and Cats

**DOI:** 10.3390/ani16101454

**Published:** 2026-05-09

**Authors:** Andrew Knight

**Affiliations:** 1 School of Veterinary Medicine, College of Environmental and Life Sciences, Murdoch University, 90 South St., Murdoch, Perth, WA 6150, Australia; andrew.knight@murdoch.edu.au; 2 School of Environment and Science, Griffith University, Nathan, Brisbane, QLD 4111, Australia; 3 Faculty of Health and Wellbeing, University of Winchester, Sparkford Road, Winchester SO22 4NR, UK; 4 Sustainable Pet Food Foundation, 147 Station Rd., London E4 6AG, UK

**Keywords:** pet food, pet diet, vegan, vegetarian, digestibility, dog, cat

## Abstract

There is growing interest in vegan and vegetarian (veg*n) diets for dogs and cats, due to factors including pet health, environmental sustainability and farmed animal welfare. Such diets should be carefully designed and manufactured in order to be nutritionally sound. Digestibility is a key, although not the only, determinant of this, and it has sometimes been claimed that dogs and cats cannot effectively digest and utilize plant-based proteins. To evaluate this claim, studies assessing canine and/or feline digestibility of veg*n diets and ingredients were analyzed. Thirty-one studies were included: 22 specific to dogs, two specific to cats, and seven applicable to both species. Across various study designs, populations, digestibility metrics, dietary ingredients and processing methods, digestibility values of veg*n diets were consistently high and broadly comparable to those of conventional meat-based diets. In all five studies that assessed apparent total tract digestibility (ATTD) of veg*n pet diets, ATTD values exceeded 80% (dry matter), 85% (organic matter), 80% (crude protein), 89% (fat), 88% (nitrogen-free extract), and 86% (energy). These studies also indicate that individual vegan protein sources can be well digested by dogs and/or cats, including those derived from legumes (such as soy-derived ingredients), pulses, grains and microbial fermentation. Discrepancies exist regarding whether these ingredients are more, less or equally digestible compared to animal-based alternatives. Nevertheless, even in studies where vegan protein sources showed lower digestibility for specific nutrients, overall digestibility remained high. These findings support the use of nutritionally sound veg*n pet diets. Such diets are not normally significantly less digestible than conventional meat-based diets.

## Simple Summary

There is growing interest in vegan and vegetarian (veg*n) pet diets among dog and cat carers concerned about the impacts of meat-based diets on farmed animals, the environment, and pet health. However, some are concerned that veg*n diets may not be adequately digested by pets who are descended from carnivores (dogs), or remain biologically carnivorous (cats). This study explores the evidence surrounding this concern by reviewing 31 relevant studies. Twenty-two were applicable to dogs, two to cats, and seven to both species. A variety of study designs, populations, digestibility measurements, dietary ingredients and processing methods were examined. Across these studies digestibility values of veg*n diets were consistently high, and broadly comparable to those of conventional meat-based diets. The overall evidence from these studies indicates that modern veg*n diets for dogs and cats, and their main protein sources, are broadly well digested by these species.

## 1. Introduction

Vegan pet foods include those made directly from plants, as well as those produced through microbial fermentation. While plant-based pet foods currently dominate the vegan pet food market, startup companies have begun to produce fermentation-derived proteins for use in pet foods. This includes US-based Calysta, which produces FeedKind, a high protein ingredient derived from the naturally occurring bacterium *Methylococcus capsulatus* [1].

In recent years, vegan and vegetarian (hereafter ‘veg*n’) diets have emerged as popular dietary choices for people across the world [2]. Correspondingly, interest in such diets for dogs and cats is growing, as guardians seek to align their companion animals’ diets with their personal choices [3]. The global vegan pet food market has been valued at $10 billion in 2020 [4] and $27 billion in 2024 [5], and is projected to more than double in value to reach $57 billion by 2034. This represents a compound growth rate of 7.8%—reportedly nearly six times faster than the traditional pet food market [6].

Ethical and environmental concerns are among the primary drivers behind the shift towards veg*n diets for humans and companion animals [3]. The potential environmental and animal welfare benefits of transitioning all pet dogs and cats, and humans, to nutritionally sound vegan diets, were previously calculated [7]. The results indicated that by 2018 at least six billion terrestrial livestock animals were consumed annually within the diets of companion dogs, along with 0.9 billion within the diets of companion cats. In comparison 71.3 billion were consumed within human diets. Billions of marine animals were also consumed by all groups. Transition of each of these groups to vegan diets could reduce greenhouse gas (GHG) emissions by more than the total GHG emissions of the UK (for dogs), New Zealand (for cats), or the entire European Union (for humans). Substantial areas of land could also be freed up, as large as Mexico (dogs), Germany (cats), or Russia and India combined (humans) [7]. A recent life cycle assessment of 31 commercially available dry dog foods in the UK—comprising plant-based, red meat-based (beef or lamb), poultry-based, and veterinary renal diets—similarly found that plant-based foods had the lowest impacts across all environmental indicators. Compared to plant-based diets, beef-based diets were associated with considerably higher GHG emissions (31.47 vs. 2.82 kg CO_2_-eq per 1000 kcal) and land use (102.15 vs. 2.73 m^2^ per 1000 kcal), and generated 14.3 times more acidifying emissions and 16.4 times more eutrophying emissions [8].

Despite these significant benefits, some guardians perceive veg*n pet diets as less palatable or as risking poorer health outcomes than conventional animal-based diets, thus limiting their wider adoption [9]. Concerns in these areas are greater for cats, who are obligate carnivores, than for dogs, who are facultative omnivores, due to key differences in nutritional requirements and related physiology (Table 1).

However, current evidence does not support such concerns about palatability or health outcomes. A large-scale prior survey of 4060 dog or cat guardians found no consistent differences in palatability between vegan diets and conventional or raw meat-based diets [13].

A growing body of research also suggests that well-formulated vegan diets can be nutritionally adequate for both dogs and cats, leading to comparable or superior health outcomes relative to conventional animal-based diets. By early 2026, at least 12 analyses had assessed the health outcomes of dogs fed veg*n diets, with 11 supporting the use of such diets [14,15,16,17,18,19], [20,21] (which utilized a single data set), [22,23,24], and only one study [25] demonstrating a contrary result. Furthermore, a recent nutritional analysis of 31 commercially available complete dry dog foods in the UK found that plant-based dog foods provide comparable nutrition to their meat-based counterparts “with respect to the majority of macro- and micronutrients, with the exception of iodine and B vitamins, which could easily be supplemented” [26].

Three credible studies [27,28,29] and one veterinary masters thesis [15] had assessed the health outcomes of veg*n diets in cats by early 2026, all of which generally supported their use. The study by Semp [15] did report subclinical deficiencies in serum folic acid in eight cats fed vegan diets, but the health of the 15 cats studied was generally good, with no diet-associated diseases detectable. Similarly, three cats partly fed table scraps (which are not nutritionally sound) among 34 cats fed vegetarian diets studied by Wakefield et al. [27] had subclinical deficiencies in plasma taurine, however no clinical signs resulted.

Additionally, a systematic review of 16 studies assessing the effects of vegan diets on the health of dogs and cats concluded, “there was no overwhelming evidence of adverse effects arising from use of these diets and there was some evidence of benefits.” [30].

Another common concern surrounding veg*n diets for companion animals is nutrient bioavailability [31], i.e., the extent to which nutrients become available to cells for use in physiological functions [32]. Bioavailability is largely determined by digestibility, which is the proportion of an ingested nutrient that is absorbed by the body. Digestibility is a particularly important consideration in the formulation of diets for animals such as dogs and cats, as they possess relatively short intestinal tracts [12]. It has been claimed that dogs and cats can more easily absorb and utilize animal-based protein than plant-based protein [33], due to the presence of anti-nutritional factors (ANF) like phytates which can impact mineral bioavailability, and trypsin inhibitors and oligosaccharides in legumes and pulses [12]. Furthermore, plant-based protein sources often contain high levels of fiber, which may inhibit lipid absorption by binding to bile acids and promoting their excretion [12].

If veg*n pet diets were significantly less digestible than conventional animal-based diets, one might expect to observe poorer health outcomes in companion animals maintained on them. However, current evidence does not suggest this is the case. Despite growing interest in veg*n diets for dogs and cats, no formal review of the scientific evidence concerning their digestibility has been conducted to date. Thus, the aim of this review was to collate and analyze evidence on canine and feline digestibility of veg*n diets and vegan ingredients.

## 2. Materials and Methods

The bibliographic databases Web of Science and Scopus were used to identify relevant studies from peer-reviewed journals, in line with best practice [34]. These bibliographic databases were selected due to their extensive coverage of the life and health sciences. Web of Science contains over 235 million records [35], and Scopus has more than 100 million, with over a quarter related to the life sciences [36].

A pilot search was conducted to determine the suitability of the search phrase. Based on this, the following search string was developed: “digestibility” AND (“pet” OR “dog” OR “cat”) AND (“vegan” OR “vegetarian” OR “plant-based” OR “meat-free”). Studies published from 2000 onwards were considered. The search was conducted between 16 June and 15 July 2025. Titles and abstracts of retrieved studies were screened for relevance, and reference lists of selected studies were reviewed to identify additional publications for inclusion.

**Figure 1:**
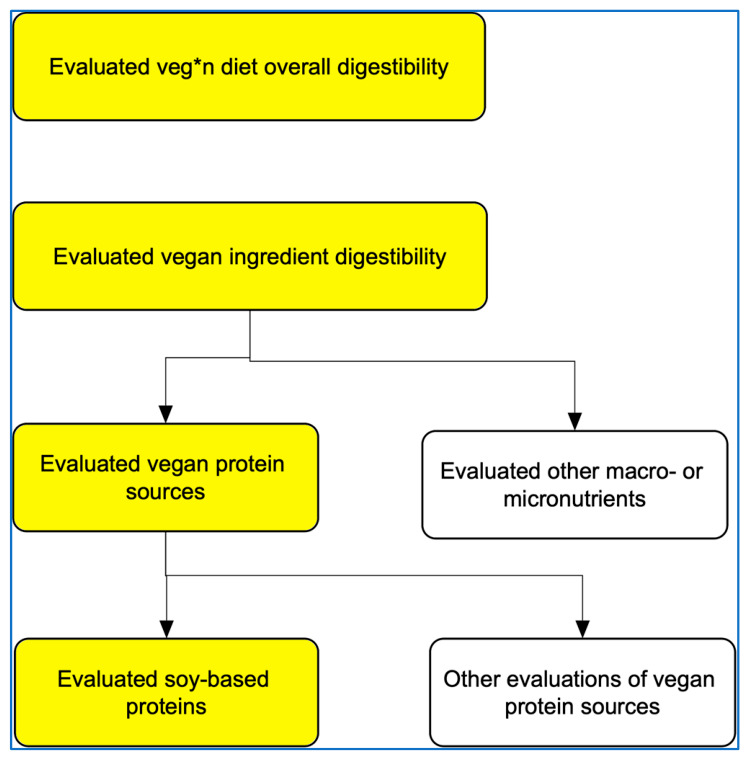
Studies examining the digestibility of veg*n dog or cat diets, or vegan ingredients. Note: Highlighted studies were analyzed.

Studies were considered for inclusion if they assessed the digestibility of fully veg*n diets or specific vegan ingredients (plant-based or derived via microbial fermentation). Given the volume of literature on vegan ingredient digestibility, the scope was narrowed to studies specifically evaluating vegan protein sources (as distinct from other macro- or micronutrients). Similarly, due to the abundance of studies comparing soy-based to animal-derived proteins, within this group of soy-based studies the analysis focused almost exclusively on those comparing soy to poultry protein—the most common protein source in pet food in the US [37] (Figure 1).

To provide a comprehensive overview of the digestibility of veg*n diets and vegan ingredients in companion animals, studies were included that assessed the digestibility of dry matter (DM) (i.e., foodstuff remaining after water extraction), organic matter (OM), fat, nitrogen-free extract (NFE) (i.e., soluble carbohydrates, starches and fermentable fiber fractions), crude protein (CP), individual amino acids (AAs), and/or energy.

Studies were excluded if they were not directly relevant to the research topic—for example, those not related to dogs or cats, those that assessed the digestibility of non-vegan protein sources (e.g., insect meal) without comparison to vegan protein sources, or those that did not directly measure digestibility. Several articles were also excluded because they were not published in English or because the full text was unavailable.

## 3. Results and Discussion

**Figure 2:**
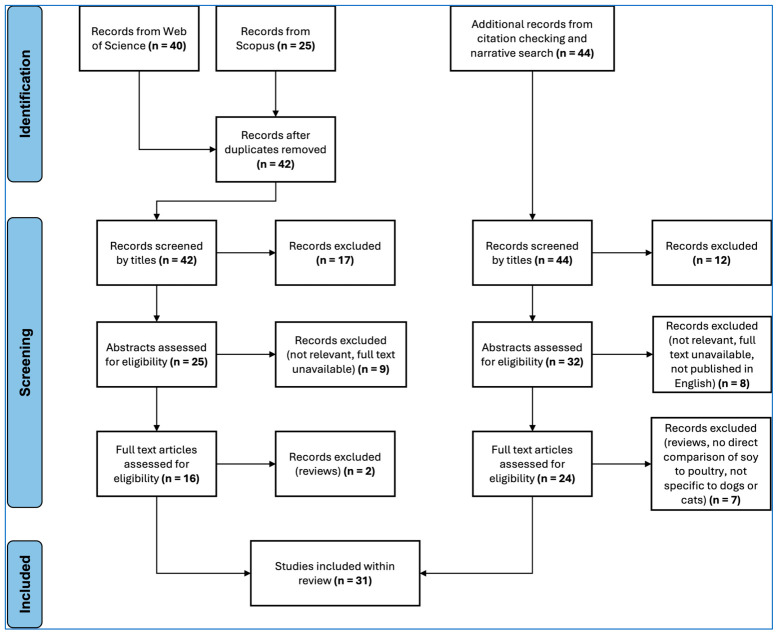
The number of studies (n) identified, screened, retained, or removed at each stage of the review process.

The search phrase returned 40 articles from Web of Science and 25 from Scopus. After removing duplicates, analyzing all search results and the reference lists of relevant articles, 31 studies met the inclusion criteria and were included in this review. The process taken to identify, screen, retain, or remove studies is illustrated in Figure 2. The 31 studies included in this review are summarized in Table S1 [1,3,14,38,39,40,41,42,43,44,45,46,47,48,49,50,51,52,53,54,55,56,57,58,59,60,61,62,63,64,65]. Twenty-two of these studies were specific to dogs, two were specific to cats, and seven were applicable to both species.

### 3.1. Digestibility Measurements

Some studies assessed total tract digestibility while others assessed ileal digestibility. Total tract digestibility measures the percentage of a nutrient or energy absorbed throughout the entire digestive tract, i.e., from ingestion to fecal excretion. Ileal digestibility calculates absorption occurring by the end of the small intestine (ileum), thus excluding digestion that occurs in the large intestine [66]. Ileal digestibility is generally considered a more accurate measure of protein digestibility than total tract digestibility, because microbial fermentation in the large intestine can alter AA concentrations in feces. In pigs, for example, it has been shown that more than 80% of AAs in feces originate from microbial activity rather than dietary protein [67]. Moreover, most if not all protein digestion occurs in the small intestine [66].

The studies also differed in whether they assessed apparent digestibility, standardized digestibility, or true digestibility. These measures vary in how they account for endogenous losses. Apparent digestibility measures the difference between what an animal consumes and what is recovered in the ileal digesta or feces, and does not correct for endogenous losses [68]. Standardized digestibility accounts for basal endogenous losses that occur irrespective of diet. True digestibility adjusts for both basal and diet-induced endogenous losses, and is therefore the most accurate estimate of the three [68].

### 3.2. Vegan or Vegetarian Diets

Seven studies assessed the digestibility of fully veg*n pet diets, six of which included comparisons with non-veg*n diets. The results of one study were applicable to both dogs and cats, while the remaining six were specific to dogs. Four studies assessed vegan diets and three assessed vegetarian diets. Five studies measured apparent total tract digestibility (ATTD) (Table S2; [3,14,53,54,58]), one measured apparent ileal digestibility (AID) in vivo, and one estimated AID using an in vitro model simulating digestion up to the end of the small intestine.

Ingenpaß et al. [54] compared the ATTD of nutrients in two extruded, isonutrient dog diets: a meat-based control (containing poultry meal (PM) and poultry fat) and a vegetarian test diet (containing wheat gluten, rice protein and sunflower oil). The vegetarian diet included vitamin D3 (0.045 g/kg diet) from lanolin in sheep’s wool, classifying it as vegetarian rather than vegan. It is worth noting that vegan versions of vitamin D3 are available, as it can be derived from lichen [69]. The two diets met the National Research Council’s (NRC) nutritional requirements for the maintenance of adult dogs. ATTD values were determined from fecal samples of six beagles that had been fed the diets for 12 days. Both diets were highly digestible, with ATTD exceeding 85% for OM, 80% for CP, 93% for fat, and 88% for NFE (Table S2). ATTD of OM, CP and fat did not differ significantly between diets, although ATTD of NFE was significantly (albeit marginally) higher in the meat-based diet (89.5%) than the vegetarian diet (88.6%) (*p* < 0.05).

Two additional studies assessed the digestibility of vegetarian dog diets. El-Wahab et al. [53] fed beagles an extruded vegetarian diet containing wheat, rice, linseed, sunflower oil and beet pulp, as well as the same diet supplemented with hydrolyzed feather meal (HFM) combined with either corn meal, rye, or fermented rye. ATTD of OM, CP and fat ranged from 83.5 to 85.8%, 76.4–80.0%, and 86.5–89.1% respectively (Table S2), with no significant differences among diets. Brown et al. [14] also found that dogs can effectively digest vegetarian diets. An extruded vegetarian diet containing maize gluten and soymeal as the main protein sources was fed to mixed-breed dogs, resulting in ATTD values of 83.1% (DM), 89.4% (CP), and 87.5% (energy) (Table S2).

These three vegetarian diet studies assessed protein digestibility based on CP, which has its limitations. The approach assumes all nitrogen in the diet originates from protein, and that all protein contains a fixed nitrogen content of 16% [70]. Furthermore, it does not account for the AA composition of the protein. Measuring the digestibility of individual AAs is more informative, as protein synthesis can be limited by the inadequate digestion of any essential amino acid (EAA) [71]. Additionally, the three studies assessed total tract rather than ileal digestibility. As discussed previously, the latter is considered a more accurate measure of protein digestibility.

These limitations were not present in a later study by Roberts et al. [61], which evaluated AID of AAs in three commercial dog diets: a chicken-based extruded diet (CT), and two mildly cooked, human-grade vegan diets. Diet BC included organic pea protein, whole peas and lentils as protein sources, while diet BR contained organic pea protein, whole peas and chickpeas. All three diets met the US Association of American Feed Control Officials’ (AAFCO) nutrient requirements for the maintenance of adult dogs. Roberts et al. [61] found that most essential and non-essential AAs had high digestibility values exceeding 80% across all diets. The only significant difference in AID of EAAs was for tryptophan, which was higher in CT than BC (*p* < 0.05). Nevertheless, tryptophan digestibility was greater than 92% in all diets.

Roberts et al. [61] measured AA digestibility using 12 cecectomized roosters, i.e., birds that had undergone surgical removal of their ceca—the pouches located at the intersection of the small and large intestines. The cecectomized rooster model is a well-accepted method of evaluating canine AA digestibility, as it avoids the confounding effects of microbial fermentation in the large intestine [61]. Digestibility values calculated using this model have been found to closely align with those from the ileal-cannulated dog model [72], which has largely fallen out of use due to animal welfare concerns. The ileal-cannulated dog model involves surgically implanting a cannula at the end of a dog’s ileum, to collect digesta before it enters the large intestine [73].

Despite the widely accepted accuracy of the cecectomized rooster model, Roberts et al. [61] acknowledged that an in vivo study in dogs would be valuable to verify their results in the target species. A subsequent study was thus conducted, assessing the ATTD of DM, OM, CP, fat and energy for the same three commercial diets (CT, BC and BR) [3]. Analysis of fecal samples from 12 beagles revealed that all three diets were highly digestible, with ATTD values surpassing 80% for all macronutrients (Table S2). ATTD of fat was slightly but significantly higher in both vegan diets than the CT (94.8% (BC) and 94.2% (BR) vs. 91.7% (CT); *p* < 0.001). However, the vegan diets had a slightly lower ATTD of OM compared to CT (85.0% (BC) and 86.3% (BR) vs. 87.1% (CT); *p* < 0.05). No significant differences were observed in the ATTD of DM, CP or energy.

The two studies by Roberts et al. [3,61] compared mildly cooked, human-grade diets with an extruded kibble diet. Any effects of ingredient composition cannot therefore be distinguished from those of the processing method. Additionally, the studies by Roberts et al. (and the three vegetarian diet studies) were conducted under controlled laboratory conditions, potentially limiting the applicability of the findings to the wider companion dog population. Such laboratory settings restrict sample size and diversity, while also raising considerable animal welfare concerns. To avoid these limitations, Liversidge et al. [58] assessed the digestibility of a vegan extruded dry kibble diet and a commercial extruded chicken-based diet in 61 healthy adult companion dogs, using in-home digestibility trials. Both diets met AAFCO nutrient requirements for adult dogs at maintenance. The vegan diet was formulated to be isoenergetic and as nutritionally similar as possible to the animal-based diet, and contained ingredients such as peas, barley, oats, potato protein, sunflower oil, lentils and quinoa. After 12 weeks of feeding, ATTD of DM, CP and fat were measured via fecal sample analysis, and exceeded 80%, 85% and 97% respectively, in both diets (Table S2). There were no significant differences in any of these measures, between diets.

Wehrmaker et al. [64] aimed to further advance understanding of the digestibility of vegan pet foods, specifically those manufactured using shear cell technology—a process of creating meat-like structures by applying heat and pressure to plant-based protein mixtures. The study compared an animal-based canned pet food to several vegan canned alternatives, all of which were wheat gluten-based and contained either pea protein isolate, soy protein isolate or faba bean concentrate. After processing via shear cell technology, the products were canned in either gravy or water and sterilized. In vitro digestion experiments were conducted to assess the digestibility of DM and CP at the end of the small intestinal phase. Digestibility of the vegan products was greater than or comparable to that of the animal-based pet food. DM digestibility was substantially higher in the vegan products, ranging from 77.3 to 81.9% compared to 56.6–57.6% in the animal-based food. CP digestibility ranged from 91.6 to 94.2% in the vegan products, and 91.7–91.8% in the animal-based product.

Across the five studies assessing the ATTD of veg*n pet diets, digestibilities were broadly similar for these diets, ranging from 80.6 to 84.7% (DM), 85.0–86.3% (OM), 88.6–89.0% (NFE), and 86.5–87.5% (energy) (Table S2). ATTD of CP and fat were more variable, ranging from 79.9 to 89.4% and 88.8–97.1% respectively. Some variability is expected given differences in study populations and environments (laboratory-housed beagles, laboratory-housed mixed-breeds and at-home companion dogs), as well as diet characteristics such as ingredient composition and processing methods. Among animal-based diets, ATTD values varied from 80.5 to 83.4% (DM), 83.5–87.0% (OM), 76.4–85.6% (CP), 86.5–97.3% (fat) and 87.5–89.5% (NFE) (Table S2). In addition to the aforementioned factors, digestibility is also affected by nutrient profile, which tends to be less consistent in animal sources than plant sources due to differences in carcass components included and in rendering processes [45,61]. This variability may also partially explain why specific digestibility metrics were sometimes higher in veg*n diets (e.g., fat digestibility in Roberts et al. [3]; DM digestibility in Wehrmaker et al. [64]), and sometimes higher in animal-based diets (e.g., OM digestibility in Roberts et al. [3]; NFE digestibility in Ingenpaß et al. [54]; tryptophan digestibility in Roberts et al. [61]) (Table S2).

Overall, the digestibility of veg*n pet diets was broadly comparable to that of animal-based diets, with all seven studies concluding that veg*n diets were well-digested. The consistency of this finding across diverse study populations, settings, nutrients, digestibility types, methodologies, and diet characteristics strengthens its reliability.

### 3.3. Vegan Protein Sources

Studies assessing the digestibility of vegan protein sources are not fully reflective of the digestibility of veg*n diets, but can nonetheless offer valuable insights. As noted previously, due to the abundance of studies in this area, those examined in this section focused almost exclusively on studies comparing soy to poultry protein.

#### 3.3.1. Soy

Soybean is the most extensively studied vegan protein source within companion animal nutrition. It has attracted much attention due to its high protein content and AA profile which closely resembles that of meat [14]. Since 2000, over 20 studies have assessed canine digestibility of soy-derived ingredients, including whole soybeans, soybean meal (SBM), soybean flour, soybean nuggets, and soybean protein concentrate (SPC) [74]. One study was identified that directly assessed the digestibility of soy protein sources in cats, and two others yielded findings applicable to both cats and dogs.

The feline-specific study, conducted by Carciofi et al. [42], used six laboratory-housed mixed-breed cats to evaluate the digestibility of an extruded diet in which micronized soybeans (whole soybeans processed using infrared heat treatment) (μSB) provided 40% of dietary protein. The diet was found to be highly digestible; ATTD values were 82% (DM), 85% (OM), 84% (CP), 90% (fat), and 86% (energy).

Two additional studies employed the cecectomized rooster model to assess standardized ileal digestibilities (SIDs) of EAAs in soy protein sources, with results considered applicable to both cats and dogs. While the model’s accuracy is more firmly established for canine than feline nutrition, differences between the two species are considered negligible when protein digestibility exceeds 90% [52,75,76]. Reilly et al. [57] reported very high SID values of EAAs in SBM (91.3–96.3%), but lower values in soy flakes (71.6–85.2%). The authors attributed this disparity to the presence of ANFs in soy flakes, which are typically denatured and inactivated through heat treatment during the production of SBM. It should be noted that most commercial pet foods are extruded [77], making it unlikely that ANFs would significantly impair the digestibility of such diets. A subsequent study by the same authors found SPC to be highly digestible, with SID of EAAs ranging from 92.0 to 96.3% [52].

Soy-based protein sources and diets have demonstrated high digestibility in dogs, with ATTD values frequently exceeding 80% for DM, OM and CP, and 90% for fat (e.g., [42,43,44,45,48,78,79,80,81,82]). High ileal digestibility has similarly been reported, with AID of EAAs in soy-based protein sources often surpassing 80% [38,39].

A number of articles have directly compared soy-based extruded dog diets to those containing poultry protein, with varied results. These studies were conducted under laboratory conditions, often using beagles (Table S3 [46,49,55,56,59,62] and Table S4 [38,39]). Two such studies—Venturini et al. [48] and Tortola et al. [43]—reported no differences in the ATTD of DM, OM, CP, fat or energy between soy-based and poultry-based diets. Venturini et al. [48] compared a diet based on poultry byproduct meal (PBPM) to diets in which SPC replaced 15%, 30% or 45% of dietary protein, and Tortola et al. [43] compared diets containing either ~30% SBM or poultry meal (PM).

Other studies have reported both positive and neutral digestibility outcomes for soy-based dog diets when compared to poultry-based diets, depending on the specific digestibility measure assessed. Menniti et al. [44] formulated diets with increasing inclusion levels of SBM (0–17%, as-fed basis), replacing up to 30% of dried chicken protein. ATTD of CP and fat increased linearly with SBM inclusion (*p* < 0.05), although no significant differences were observed in ATTD of DM or OM among diets. Another study measured ATTD and AID in diets containing either SBM, soy flour, SPC, or PM [39]. While the ATTD and AID of DM, OM, fat and energy did not differ significantly between soy and poultry diets, both AID and ATTD of CP were higher in the soy diets (*p* < 0.01) (Table S4). In addition, AID of all EAAs were higher in the soy diets (*p* < 0.05), except for methionine and threonine, which showed no significant differences between treatments [39]. Carciofi et al. [42] compared three isonutrient diets containing either μSB, SBM or PBPM, and found that the ATTD of DM and fat was significantly higher in the μSB diet than the PBPM diet (*p* < 0.05). No significant differences were detected in ATTD of OM, CP or energy between diets.

Conversely, some studies have observed both negative and neutral digestibility outcomes for soy-based diets compared to poultry-based diets in dogs. Yamka et al. [41] reported that ATTD of DM was on average significantly higher in PBPM diets (91.3%) than SBM diets (87.0%), although ATTD of CP was similar. Beloshapka et al. [45] also found no difference in ATTD of CP among diets containing increasing levels of bioprocessed soy protein (BSP) in place of PBPM (0–48% BSP inclusion). ATTD of DM, OM, fat and energy remained unaffected up to 24% BSP, but declined significantly at 48% BSP (*p* < 0.01). Bednar et al. [38] measured both AID and ATTD in diets containing SBM, PM, PBPM, or beef and bone meal (BBM). AID of DM, OM, fat and CP were similar across diets (Table S4), as was ATTD of fat. However, ATTD of DM was lowest in the SBM (78.3%) and BBM (78.6%) diets compared to the PM (84.5%) and PBPM (83.0%) diets (*p* < 0.05). ATTD of OM was also lowest in the SBM diet (82.7%) compared to an average of 87.4% across the other diets (*p* < 0.05). While ATTD of CP was highest in the PM diet (87.5%) (*p* < 0.05), it remained relatively high in the SBM diet (82.7%). As previously noted, AID, which did not differ for CP among diets, is generally considered a more accurate measure of CP digestibility than ATTD. The authors concluded that all diets were “well utilized” and that “both plant and animal protein sources can provide adequate levels of highly digestible nutrients to the dog” [38].

One study reported positive, negative and neutral digestibility outcomes for soy compared to poultry, in dogs. Yamka et al. [40] evaluated diets based on whole soybeans, SBM or PM. AID of DM was highest in the PM diet (86.2%), compared to an average of 78.4% across the soy-based diets (*p* < 0.01). ATTD of DM and CP were also significantly higher in the PM diet, although these values were greater than 80% and 81% in all diets respectively. AID of CP did not differ among diets, while AID of several AAs (phenylalanine, cysteine, aspartate, glutamate, tyrosine, and serine) was lowest in the PM diet (*p* < 0.05).

Across studies, macronutrient and energy digestibilities were often comparable between soy-based and poultry-based dog diets, although this was not consistently observed for any single measure. Such inconsistencies likely reflect dietary differences, including types of ingredients, their nutritional profiles and inclusion levels. Nevertheless, even in studies where poultry protein showed higher digestibility for certain measures, soy-based diets were still well digested. Across all studies, ATTD values in soy diets ranged from 77.5 to 89.1% (DM), 81.7–89.2% (OM), 80.9–89.3% (CP), 88.4–95.5% (fat), and 83.8–89.6% (energy). AID of CP in soy diets ranged from 79.2 to 87.2% (Table S4).

#### 3.3.2. Other Vegan (Including Fermented Animal-Based) Protein Sources

Canine and feline digestibility has also been assessed for a range of vegan protein sources other than soy, including those derived from pulses, grains, and microbial fermentation.

Several studies have measured the digestibility of vegan protein sources using the cecectomized rooster model, with their results considered applicable to both cats and dogs. Reilly et al. [51] determined SID of EAAs in five pulse ingredients: green lentils, black bean grits, yellow peas, navy bean powder, and chickpeas. All EAAs were highly digestible across all ingredients (80.6–94.8%), except for methionine, which ranged from 72.9 to 79.1% in green lentils, black bean grits, and yellow peas. Expanding on this work, Reilly et al. [52] evaluated additional vegan protein sources, including pea protein, faba bean protein, potato protein, and dried yeast. All ingredients were well digested, with SID of EAAs ranging from 86.1 to 96.3%. In a further study, Reilly et al. [57] assessed plant-based byproducts including corn gluten meal (CGM) and peanut flour. Both ingredients exhibited high SID of EAAs, with the exception of lysine in peanut flour, which was significantly lower at 45.5% (*p* < 0.05). Building on this body of work, Oba et al. [60] directly compared the SID of AAs in several vegan ingredients (a novel microbial protein (FeedKind), pea protein, and CGM) and non-vegan ingredients (chicken meal and black soldier fly larvae). All EAA digestibilities exceeded 85% for FeedKind, and 80% for all other ingredients. CGM generally had the highest AA digestibilities while chicken meal had the lowest.

FeedKind was the first of its kind microbial protein used to produce a nutritionally complete pet diet, with the first product (nutritionally complete dog food) released in 2025 [83]. The protein is derived from cultivating a harmless bacterium, *Methylococcus capsulatus*, and has also been shown to be well digested by dogs in direct feeding trials. Longshaw et al. [1] fed dogs isocaloric, isonitrogenous and isolipidic diets for six months, containing 0%, 4%, 6% or 8% FeedKind, primarily replacing SBM. Macronutrient and energy digestibilities were similar across diet groups. For all diets, ATTD of OM, CP and energy exceeded 80%, while ATTD of fat exceeded 90% [1].

Several studies assessing canine digestibility of vegan and animal-derived protein sources have reported similar digestibility between them. Reilly et al. [55] fed dogs isocaloric and isonitrogenous diets containing either PBPM, chickpeas, peanut flour, green lentils or dried yeast as the main protein source. All diets were highly digestible, with similar ATTD of macronutrients; all ATTD values exceeded 80% for DM, OM and CP, and 85% for fat (Table S5 [46,49,55,56,59,62,65]). Another study based on data from 226 dogs found no impact on true total tract digestibility as dietary protein shifted from animal-based to plant-based sources [50].

Other studies have observed equal or superior canine total tract digestibility for vegan proteins compared to animal-based proteins. Cargo-Froom et al. [49] compared two canine diets based on predominantly animal protein (Diet one) or plant protein (Diet two). Diet two showed significantly higher ATTD of DM, OM, alanine, and leucine, as well as significantly higher true total tract digestibility of DM. No significant differences were observed in ATTD of any other AAs, CP, or fat, or in true total tract digestibility of CP ([49], Table S5). Sieja et al. [62] further demonstrated that the digestibility of certain plant proteins can equal or exceed that of animal proteins. Dogs fed a wheat gluten meal (WGM) diet had significantly higher ATTD of CP than those fed diets based on deboned chicken, PBPM, or CGM (*p* < 0.0001). ATTD of CP, OM, and energy were significantly lower in the PBPM group, and DM ATTD was significantly higher in the WGM and deboned chicken groups (*p* < 0.0001). ATTD of fat did not differ among diet groups. Urrego et al. [46] similarly reported superior digestibility outcomes for WGM compared to animal protein. French bulldogs were fed four extruded isonutrient diets, each containing 13% protein from PM; WGM; an equal mix of PM and WGM; or an equal mix of PM, WGM and liver hydrolyzed protein (LHP). The four diets were well digested; all ATTD values were greater than 85% (DM), 88% (OM), 83% (CP), 96% (fat), 91% (NFE) and 88% (energy) (Table S5). The diets containing WGM had significantly higher ATTD of DM, OM and CP, compared to the others (*p* < 0.05).

French et al. [65] evaluated the digestibility of a lamb protein produced through precision fermentation (PF), a technology which involves culturing genetically engineered micro-organisms in bioreactors with a carbon-based feedstock, which is converted into specific ingredients [83]. Dogs were fed diets formulated with 0% (control), 15%, 30%, or 40% of the PF lamb protein, replacing 0%, 37%, 75%, or nearly 100% (99.6%) of the egg protein respectively. The authors reported a mix of positive, negative, and neutral digestibility outcomes for the PF protein compared to egg protein. Apparent and true total tract digestibility of protein showed no significant differences and remained high across all diets, exceeding 86% and 91% respectively. However, ATTD of fat was significantly lower in the PF lamb protein diets (81.0–85.9%) compared to the control (92.4%). In contrast, ATTD of DM was significantly higher in the 30% and 40% PF lamb protein diets (83.2% and 85.0% respectively) than in the control (79.3%).

Studies assessing canine ileal digestibility have reported comparable or superior digestibility for vegan proteins relative to animal-derived proteins. Fiacco et al. [47] compared three canine diets: one based on PM, and two in which approximately half the PM was substituted for vegetable protein (predominantly CGM), formulated either on a total protein (DPR) or amino acid (DAA) basis. Diets were fed to broiler chicks for eight days, after which ileal digesta were collected to assess AID. AIDs of most AAs were significantly higher in birds fed the vegetable protein DPR and/or DAA diets compared to the PM diet.

Hsu et al. [63] conducted a more comprehensive analysis of the ileal AA digestibilities of vegan versus animal-derived protein sources. Eight dog diets were formulated, containing predominately animal or plant-based protein ingredients: PBPM; chicken slurry; chicken slurry and yellow peas; yellow peas; green lentils; chickpeas; chicken slurry and taurine; and chicken slurry and yellow peas and taurine. Two versions of each diet were prepared, one extruded using a single screw extruder and the other using a twin screw extruder. Digestibility was measured at three processing stages: the raw ingredient mix, after preconditioning, and at the extruder exit. At the final stage (wet kibbles exiting the extruder), all SIDs of EAAs exceeded 80% across all diets, except the three that lacked pulse ingredients: PBPM (both extruders), chicken slurry (twin screw extruder), and chicken slurry and taurine (twin screw extruder). All SIDs of EAAs were significantly lower in the chicken slurry and taurine diet (twin screw extruder) than in all other formulations (*p* < 0.05). The authors also found that extrusion significantly increased EAA digestibility; all diets had some SIDs of EAAs below 80% at the raw ingredient stage [63].

Like dogs, cats have also demonstrated the ability to effectively digest various vegan protein sources, although relevant studies are fewer. In a study by Morris et al. [59], cats were fed diets containing increasing levels of rice protein concentrate (RPC) (0%, 7%, 14%, and 28%), replacing hydrolyzed chicken liver and heart. All plant-based diets were highly digestible, with ATTD values surpassing 80% (DM), 90% (fat), 89% (NFE) and 83% (energy), while true total tract protein digestibility was above 90% (Table S5). Moreover, all measures increased linearly in proportion to RPC inclusion (*p* < 0.05), except for fat digestibility, which remained unchanged.

Golder et al. [50] similarly observed digestibility advantages of plant-based protein over animal-based protein. In their study of 296 cats, true total tract protein digestibility increased as the proportion of dietary protein from plant sources increased; a 5.5% increase was observed at 50% plant protein in dry cat food [50]. The study also assessed individual plant protein ingredients, and found that increasing protein from CGM had a significant positive effect on protein digestibility. Carciofi et al. [42] also demonstrated that cats can efficiently digest CGM. In a diet where CGM constituted 40% of dietary protein, ATTD values were 81% (DM), 85% (OM), 84% (CP), 86% (fat), and 85% (energy). Reilly et al. [56] showed that cats are also capable of digesting legumes. In cats fed extruded diets formulated with 0–30% chickpeas (replacing PBPM and rice), ATTD of DM, OM, CP and fat exceeded 81%, 86%, 82% and 92% respectively when chickpeas were raw. These values fell slightly when chickpeas were cooked. Raw chickpea inclusion level did not impact digestibility (Table S5).

The evidence reviewed suggests that both dogs and cats can effectively digest a variety of vegan protein sources, although it must be acknowledged that studies in dogs are considerably more numerous than those in cats. In many cases, their digestibility values are comparable with or greater than those of conventional animal-derived protein sources (Table S5), thus supporting the inclusion of these vegan protein sources within nutritionally complete pet foods. However, proper processing of dietary ingredients is essential to maximize nutrient availability. Some under-processed ingredients (e.g., raw pulses) contain ANFs that can reduce the digestibility of certain AAs, such as methionine [51,63]. Conversely, heat processing can decrease the digestibility of other AAs by triggering Maillard reactions—non-enzymatic chemical reactions between the carbonyl groups of reducing sugars and the amino groups of amino acids, which result in the formation of non-bioavailable compounds [84]. Lysine is particularly susceptible to Maillard reactions due to the ε-amino group in the side chain; Reilly et al. [57] attributed the low digestibility of lysine in peanut flour to Maillard reactions resulting from the peanut roasting process. Pet food manufacturers should be aware of these nuances, and should optimize processing conditions for the specific ingredients used. Where necessary, complementary protein sources should be included to ensure that reduced digestibility of a particular AA in one ingredient does not impair overall protein synthesis.

### 3.4. Limitations and Future Research Suggestions

While current evidence suggests that well-formulated veg*n pet diets are highly digestible, further research would be beneficial in some areas. For example, the studies referenced generally applied to adults fed maintenance diets. They did not examine the suitability of veg*n diets for puppies, kittens, senior animals, or individuals with compromised digestive function. Additionally, most (22) existing studies were specific to dogs, with fewer (two) specific to cats, or applicable to both species (seven). Most studies were also conducted under controlled, laboratory conditions, which may limit the generalizability of their findings to the broader population of companion animals [58]. Future research assessing the digestibility of veg*n diets through in-home studies, particularly in cats, and to other groups such as juvenile, senior, or unwell animals, would therefore be valuable to better understand how these diets perform, including within real-world settings.

It is also important to acknowledge that this study focused on the digestibility of DM, OM, fat, NFE, CP, AAs and energy. It did not consider the digestibility of micronutrients, some of which are naturally more abundant in animal sources—including certain B vitamins, taurine, betaine, and choline. The balance of some micronutrients can also be critical. Most existing digestibility studies have focused on macronutrients rather than micronutrients, and hence additional research on the latter is warranted.

As noted, within the vegan ingredient digestibility section, the scope was limited to vegan protein sources, due to the volume of literature. This was also the reason the soy section focused almost exclusively on studies comparing soy to poultry protein—the most common protein source in pet food in the US. Future research could assess the digestibility of non-protein vegan ingredients, non-soy vegan protein sources, or comparisons to meat-based protein sources other than poultry.

It should also be acknowledged that protein digestibility alone does not fully indicate nutritional completeness. Vitamins, minerals, and other nutrients must be assessed independently, and the anti-nutritional factors associated with increased plant inclusion—such as trypsin inhibitors, phytates, and lectins—require explicit consideration.

Additionally, this review was not conducted as a systematic review. A systematic review could help ensure a more comprehensive coverage of the existing literature on the digestibility of veg*n pet diets and vegan ingredients.

## 4. Conclusions

As environmental and ethical concerns surrounding conventional animal-based diets are growing, so too is interest in veg*n diets for companion animals. This review included 31 studies on canine and/or feline digestibility of veg*n diets or vegan ingredients. Across a range of study designs, populations, digestibility metrics, dietary ingredients and processing methods, digestibility values of veg*n diets were consistently high and broadly comparable to those of conventional meat-based diets.

Current evidence also indicates that a variety of vegan protein sources can be well digested by dogs and cats—although it must be acknowledged that studies in cats are presently far fewer than those in dogs. Such vegan protein sources include those derived from legumes (such as soy-derived ingredients), pulses, grains and microbial fermentation. Some discrepancies exist regarding whether these ingredients are more, less or equally digestible compared to animal-based alternatives. Such variation is unsurprising given differences in ingredient types, processing methods and nutritional profiles. The latter are less consistent in animal-based protein sources due to variability in carcass components and rendering processes. Importantly, even in studies where certain vegan protein sources showed lower digestibilities for specific nutrients, digestibility remained high overall.

These findings support the use of nutritionally sound veg*n pet diets, and challenge the notion that such diets are inherently less digestible than conventional animal-based diets. Nevertheless, further research involving cats, and in real-world, in-home settings would be beneficial.

## Figures and Tables

**Table 1:** Key differences between dogs and cats in nutritional requirements and related physiology [10,11,12].

Feature	Dogs	Cats
Protein requirement	Moderate	High
Essential amino acids	10	Same 10 plus taurine = 11
Enzyme regulation	Adaptive	Fixed high catabolism
Pancreatic amylase	High	Low
Gluconeogenesis from amino acids	Regulated	Constant

## Data Availability

No new data were created or analyzed in this study. Data sharing is not applicable to this article.

## Supplementary Materials

The following supporting information can be downloaded at: https://www.mdpi.com/article/10.3390/ani16101454/s1, Table S1. Studies included in the review (n = 31); Table S2. Studies identified (n = 5) assessing apparent total tract digestibility (ATTD) of macronutrients and energy in fully vegan or vegetarian (‘veg*n’) versus animal-based dog diets; Table S3. Studies identified (n = 6) comparing apparent total tract digestibility (ATTD) of macronutrients and energy in dog diets formulated with specific vegan ingredients versus poultry-based ingredients; Table S4. Studies identified (n = 2) comparing apparent ileal digestibility (AID) of macronutrients and energy in soy-based versus animal-based dog diets; Table S5. Studies identified (n = 7) comparing apparent total tract digestibility (ATTD) of macronutrients and energy in diets formulated with specific vegan (including fermented animal-based) ingredients versus animal-based diets for dogs or cats.
