# Supplementary material for: The Digestibility of Vegan and Vegetarian Diets for Dogs and Cats
Source: Animals (Basel). 2026 May 9;16(10):1454. doi: 10.3390/ani16101454 (PMC13203682; doi:10.3390/ani16101454)
Supplement: Supplementary file 1 [file animals-16-01454-s001.zip › animals-4267664-supplementary.pdf]

Supplementary Material

Table S1. Studies included in the review (n=31).

	Author(s)	Year	Study title	Target species
1	Bednar *et al.* [38]	2000	Selected animal and plant protein sources affect nutrient digestibility and fecal characteristics of ileally cannulated dogs	Dog
2	Clapper *et al.* [39]	2001	Ileal and total tract nutrient digestibilities and fecal characteristics of dogs as affected by soybean protein inclusion in dry, extruded diets	Dog
3	Yamka *et al.* [40]	2005	Evaluation of low-oligosaccharide and low-oligosaccharide low-phytate whole soya beans in canine foods	Dog
4	Yamka *et al.* [41]	2006	In vivo measurement of flatulence and nutrient digestibility in dogs fed poultry by-product meal, conventional soybean meal, and low-oligosaccharide low-phytate soybean meal	Dog
5	Brown *et al.* [14]	2009	An experimental meat-free diet maintained haematological characteristics in sprint-racing sled dogs	Dog
6	Carciofi *et al.* [42]	2009	Comparison of micronized whole soybeans to common protein sources in dry dog and cat diets	Dog and cat
7	Tortola *et al.* [43]	2013	Enzyme effects on extruded diets for dogs with soybean meal as a substitute for poultry by-product meal	Dog
8	Menniti *et al.* [44]	2014	Effect of graded inclusion of dietary soybean meal on nutrient digestibility, health, and metabolic indices of adult dogs	Dog
9	Beloshapka *et al.* [45]	2016	Apparent total tract macronutrient digestibility, fecal characteristics, and fecal fermentative end-product concentrations of healthy adult dogs fed bioprocessed soy protein	Dog
10	Urrego *et al.* [46]	2017	Effects of different protein sources on fermentation metabolites and nutrient digestibility of brachycephalic dogs	Dog

11	Fiacco *et al.* [47]	2018	Evaluation of vegetable protein in canine diets: assessment of performance and apparent ileal amino acid digestibility using a broiler model	Dog
12	Venturini *et al.* [48]	2018	Processing traits and digestibility of extruded dog foods with soy protein concentrate	Dog
13	Cargo-Froom *et al.* [49]	2019	Apparent and true digestibility of macro and micro nutrients in adult maintenance dog foods containing either a majority of animal or vegetable proteins	Dog
14	Golder *et al.* [50]	2020	Cats have increased protein digestibility as compared to dogs and improve their ability to absorb protein as dietary protein intake shifts from animal to plant sources	Dog and cat
15	Reilly *et al.* [51]	2020a	Macronutrient composition, true metabolizable energy and amino acid digestibility, and indispensable amino acid scoring of pulse ingredients for use in canine and feline diets	Dog and cat
16	Reilly *et al.* [52]	2020b	Use of precision-fed cecectomized rooster assay and digestible indispensable amino acid scores to characterize plant- and yeast-concentrated proteins for inclusion in canine and feline diets	Dog and cat
17	El-Wahab *et al.* [53]	2021	Nutrient digestibility of a vegetarian diet with or without the supplementation of feather meal and either corn meal, fermented rye or rye and its effect on fecal quality in dogs	Dog
18	Ingenpaß *et al.* [54]	2021	Nitrogen output in the urban environment using a vegetarian canine diet	Dog
19	Reilly *et al.* [55]	2021a	Use of legumes and yeast as novel dietary protein sources in extruded canine diets	Dog
20	Reilly *et al.* [56]	2021b	Effects of graded inclusion levels of raw garbanzo beans on apparent total tract digestibility, fecal quality, and fecal fermentative end-products and microbiota in extruded feline diets	Cat
21	Reilly *et al.* [57]	2021c	Use of the precision-fed cecectomized rooster assay to determine standardized amino acid digestibility, true metabolizable energy content, and digestible indispensable amino acid scores of plant-based protein by-products used in canine and feline diets	Dog and cat
22	Liversidge *et al.* [58]	2023	Extruded diet macronutrient digestibility: plant-based (vegan) vs. animal-based diets in client-owned healthy adult dogs and the impact of guardian compliance during in-home trials	Dog

23	Morris *et al.* [59]	2023	Rice protein concentrate is a well-accepted, highly digestible protein source for adult cats	Cat
24	Oba *et al.* [60]	2023	Comparing the standardized amino acid digestibility of an alternative protein source with commercially available protein-based ingredients using the precision-fed cecectomized rooster assay	Dog and cat
25	Roberts *et al.* [3]	2023a	Apparent total tract macronutrient digestibility of mildly cooked human-grade vegan dog foods and their effects on the blood metabolites and fecal characteristics, microbiota, and metabolites of adult dogs	Dog
26	Roberts *et al.* [61]	2023b	Amino acid digestibility and nitrogen-corrected true metabolizable energy of mildly cooked human-grade vegan dog foods using the precision-fed cecectomized and conventional rooster assays	Dog
27	Sieja *et al.* [62]	2023	Evaluation of high-protein diets differing in protein source in healthy adult dogs	Dog
28	Hsu *et al.* [63]	2024	Effects of single and twin thermal screw extrusion on protein quality of grain-free pet foods formulated with predominantly animal- or plant-based protein ingredients	Dog
29	Wehrmaker *et al.* [64]	2024	In vitro digestibility and solubility of phosphorus of three plant-based meat analogues	Dog and cat
30	French *et al.* [65]	2025	Safety and digestibility of a novel ingredient, brewed lamb protein, in healthy adult dogs	Dog
31	Longshaw *et al.* [1]	2025	The safety of FeedKind Pet® (*Methylococcus capsulatus,* Bath) as a cultured protein source in the diet of adult dogs and its effect on feed digestibility, fecal microbiome, and health status	Dog

Table S2. Studies identified (n=5) assessing apparent total tract digestibility (ATTD) of macronutrients and energy in fully vegan or vegetarian ('veg*n') versus animal-based dog diets.
Note: DM=dry matter, OM=organic matter, CP=crude protein, NFE=nitrogen-free extract. Digestibility data are highlighted in green for veg*n diets and red for animal-based diets.

	**Brown *et al.* (2009)** [14]	**El-Wahab *et al.* (2021)** [53]	**Ingenpaß *et al.* (2021)** [54]	**Roberts *et al.* (2023a)** [3]	**Liversidge *et al.* (2023)** [58]
**Veg*n diet(s)**	Extruded vegetarian diet containing maize gluten and soymeal as the main protein sources	Extruded vegetarian diet containing wheat, wheat gluten, broken rice, rice protein, linseed, sunflower oil and beet pulp ('basic diet')	Extruded vegetarian diet containing wheat gluten, rice protein and sunflower oil	Two mildly cooked human-grade vegan dog foods, one containing protein from pea protein, whole peas and lentils (BC), and the other from pea protein, whole peas and chickpeas (BR)	Extruded vegan diet containing peas, barley, oats, potato protein, sunflower oil, lentils and quinoa
**Animal-based diet(s)**	N/A	Basic diet supplemented with hydrolyzed feather meal and either corn meal (HFM+CM), rye (HFM+R), or fermented rye (HFM+FR)	Extruded meat-based diet containing poultry meal and poultry fat	Extruded chicken-based diet	Extruded chicken-based diet

Study population and design			8 laboratory-housed mixed-breed dogs	8 laboratory-housed beagles (replicated 4x4 Latin square design)	6 laboratory-housed beagles (crossover design)	12 laboratory-housed beagles (replicated 3×3 Latin square design)	61 companion dogs (vegan: n=31, chicken-based: n=30)
**Overall ranges**			**Individual study ranges**				
**Veg*n diet(s)**	**DM ATTD (%)**	**80.6-84.7**	83.1	N/A	N/A	83.2 (BC); 84.7 (BR)	80.6
	**OM ATTD (%)**	**85.0-86.3**	N/A	85	85.2	85.0 (BC); 86.3 (BR)	N/A
	**CP ATTD (%)**	**79.9-89.4**	89.4	79.9	80.3	84.4 (BC); 85.9 (BR)	85
	**Fat ATTD (%)**	**88.8-97.1**	N/A	88.8	93.5	94.8 (BC); 94.2 (BR)	97.1
	**NFE ATTD (%)**	**88.6-89.0**	N/A	89	88.6	N/A	N/A
	**Energy ATTD (%)**	**86.5-87.5**	87.5	N/A	N/A	86.5 (BC); 87.2 (BR)	N/A
**Animal-based diet(s)**	**DM ATTD (%)**	**80.5-83.4**	N/A	N/A	N/A	83.4	80.5
	**OM ATTD (%)**	**83.5-87.1**	N/A	84.1 (HFM+CM); 85.8 (HFM+FR); 83.5 (HFM+R)	86.3	87.1	N/A
	**CP ATTD (%)**	**76.4-85.6**	N/A	79.1 (HFM+CM); 80.0 (HFM+FR); 76.4 (HFM+R)	82.3	84.7	85.6
	**Fat ATTD (%)**	**86.5-97.3**	N/A	88.0 (HFM+CM); 89.1 (HFM+FR); 86.5 (HFM+R)	94	91.7	97.3
	**NFE ATTD (%)**	**87.5-89.5**	N/A	87.5 (HFM+CM); 89.4 (HFM+FR); 87.7 (HFM+R)	89.5	N/A	N/A
	**Energy ATTD (%)**	**87**	N/A	N/A	N/A	87	N/A

Table S3. Studies identified (n=6) comparing apparent total tract digestibility (ATTD) of macronutrients and energy in dog diets formulated with specific vegan ingredients versus poultry-based ingredients.

Note: DM=dry matter, OM=organic matter, CP=crude protein. Digestibility data are highlighted in green for veg*n diets and red for animal-based diets.

	**Urrego *et al.* (2017)** [46]	**Cargo-Froom *et al.* (2019)** [49]	**Reilly *et al.* (2021a)** [55]	**Reilly *et al.* (2021b)** [56]	**Sieja *et al.* (2023)** [62]	**Morris *et al.* (2023)** [59]
**Diet(s) formulated with specific vegan ingredients**	Extruded diet containing 13% protein from wheat gluten meal (WGM)	Extruded diet based on vegetable protein, including corn gluten meal and peas	Extruded diets containing either chickpeas (C), peanut flour (PF), green lentils (GL) or dried yeast (Y) as the main protein source	Extruded diets containing chickpea inclusion levels of 7.5% (R7.5), 15% (R15), or 30% raw chickpeas (R30), or 30% cooked chickpeas (C30)	Extruded diets containing WGM or corn gluten meal (CGM) as the protein source	Extruded diets containing rice protein concentrate (RPC) inclusion levels of 7% (R7),14% (R14) or 28% (R28)

**Poultry-based diet(s)**			Extruded diets containing 13% protein from poultry meal (PM), an equal mix of PM and WGM (PM+WGM), or an equal mix of PM, WGM and liver hydrolyzed protein (PM+WGM+LHP)	Extruded diet based on animal protein, including fresh beef, chicken, fish blend, whole egg and herring meal	Extruded diet containing poultry byproduct meal (PBPM) as the main protein source	Extruded diet based on PBPM and rice (0% chickpeas)	Extruded diets containing deboned, dried, and spray-dried chicken (DC) or PBPM as the protein source	Extruded diet containing hydrolyzed chicken liver and heart (0% RPC)
**Study population and design**			8 laboratory-housed French Bulldogs (replicated 4x4 Latin square design)	8 laboratory-housed beagles (replicated 4×4 Latin square design)	10 laboratory-housed beagles (replicated 5×5 Latin square design)	10 laboratory-housed domestic shorthair cats (replicated 5x5 Latin square design)	12 laboratory-housed beagles (replicated 4×4 Latin square design)	24 laboratory-housed domestic shorthair cats (replicated 4×4 Latin square design)
**Overall ranges**								
**Diet(s) formulated with specific vegan ingredients**	**DM ATTD (%)**	**80.1-90.7**	87.4	90.7	83.7 (C); 82.1 (GL); 85.6 (PF); 80.1 (Y)	83.7 (R7.5); 81.5 (R15); 81.4 (R30); 77.3 (C30)	85.4 (WGM); 84.6 (CGM)	82.7 (R7); 84.3 (R14); 85.1 (R28)
	**OM ATTD (%)**	**81.7-92.2**	89.6	92.2	87.5 (C); 85.8 (GL); 90.3 (PF); 84.3 (Y)	88.6 (R7.5); 86.0 (R15); 86.4 (R30); 81.7 (C30)	90.0 (WGM); 88.3 (CGM)	N/A

	**CP ATTD (%)**	**81.5-93.8**	88.1	92.2	83.2 (C); 81.5 (GL); 85.0 (PF); 83.7 (Y)	86.2 (R7.5); 82.3 (R15); 84.5 (R30); 81.6 (C30)	93.8 (WGM); 90.1 (CGM)	N/A
	**Fat ATTD (%)**	**87.9-98.0**	96.5	98	94.2 (C); 94.1(GL); 95.5 (PF); 87.9 (Y)	93.3 (R7.5); 92.8 (R15); 92.4 (R30); 91.5 (C30)	96.0 (WGM); 95.9 (CGM)	91.0 (R7); 91.7 (R14); 89.8 (R28)
	**NFE ATTD (%)**	**89.0-92.8**	92.1	N/A	N/A	N/A	N/A	89.0 (R7); 90.5 (R14); 92.8 (R28)
	**Energy ATTD (%)**	**86.0-91.0**	89.6	N/A	N/A	N/A	91.0 (WGM); 89.5 (CGM)	86.0 (R7); 87.0 (R14); 87.8 (R28)
**Poultry-based diet(s)**	**DM ATTD (%)**	**80.1-88.8**	85.2 (PM); 88.8 (PM+WGM); 86.2 (PM+WGM+LHP)	86.9	86.3	82.8	85.7 (DC); 81.6 (PBPM)	80.1
	**OM ATTD (%)**	**85.8-91.2**	88.1 (PM); 90.4 (PM+WGM); 88.3 (PM+WGM+LHP)	89.2	91.2	87.6	89.7 (DC); 85.8 (PBPM)	N/A
	**CP ATTD (%)**	**82.6-89.9**	84.0 (PM); 88.5 (PM+WGM); 83.6 (PM+WGM+LHP)	88.3	86.6	83.9	89.9 (DC); 82.6 (PBPM)	N/A
	**Fat ATTD (%)**	**91.0-97.1**	96.8 (PM); 96.9 (PM+WGM); 96.6 (PM+WGM+LHP)	97.1	94.7	93.6	96.6 (DC); 96.1 (PBPM)	91
	**NFE ATTD (%)**	**83.5-93.3**	91.7 (PM); 93.3 (PM+WGM); 91.8 (PM+WGM+LHP)	N/A	N/A	N/A	N/A	83.5

	**Energy ATTD (%)**	**83.6-90.6**	88.3 (PM); 90.2 (PM+WGM); 89.7 (PM+WGM+LHP)	N/A	N/A	N/A	90.6 (DC); 86.8 (PBPM)	83.6

Table S4. Studies identified (n=2) comparing apparent ileal digestibility (AID) of macronutrients and energy in soy-based versus animal-based dog diets.
Note: DM=dry matter, OM=organic matter, CP=crude protein. Digestibility data are highlighted in green for soy-based diets and red for animal-based diets.

			**Bednar *et al.* (2000)** [38]	**Clapper *et al.* (2001)** [39]
**Soy-based diet(s)**			Extruded diet based on soybean meal (SBM)	Extruded diets containing either SBM, soy flour (SF), traditional soy protein concentrate (SPC1), extruded SPC (SPC2) or modified molecular weight SPC (SPC3)
**Animal-based diet(s)**			Extruded diets based on poultry meal (PM), poultry byproduct meal (PPBM), or beef and bone meal (BBM)	Extruded diet based on PM
**Study population and design**			4 laboratory-housed ileal-cannulated dogs (4x4 Latin square design)	6 laboratory-housed ileal-cannulated dogs (6x6 Latin square design)
**Overall ranges**			**Individual study ranges**	
**Soy-based diet(s)**	**DM AID (%)**	**69.0-78.4**	69	73.5 (SBM); 78.4 (SF); 70.0 (SPC1); 74.0 (SPC2); 75.7 (SPC3)
	**OM AID (%)**	**73.6-80.9**	73.6	77.5 (SBM); 80.9 (SF); 75.1 (SPC1); 78.4 (SPC2); 79.9 (SPC3)
	**CP AID (%)**	**79.2-87.2**	79.2	85.3 (SBM); 87.2 (SF); 82.6 (SPC1); 84.5 (SPC2); 85.9 (SPC3)
	**Fat AID (%)**	**89.2-95.9**	89.2	94.5 (SBM); 95.9 (SF); 93.9 (SPC1); 95.0 (SPC2); 94.0 (SPC3)
	**Energy ATTD (%)**	**80.0-85.0**	N/A	81.6 (SBM); 85.0 (SF); 80.0 (SPC1); 82.8 (SPC2); 83.3 (SPC3)
**Animal-based diet(s)**	**DM AID (%)**	**65.7-79.7**	67.8 (PM); 79.7 (PBPM); 65.7 (BBM)	76.3
	**OM AID (%)**	**74.4-83.4**	74.4 (PM); 83.4 (PBPM); 75.6 (BBM)	81.4
	**CP AID (%)**	**68.3-77.2**	73.9 (PM); 77.2 (PBPM); 68.3 (BBM)	72.7
	**Fat AID (%)**	**89.2-94.8**	89.2 (PM); 92.9 (PBPM); 89.3 (BBM)	94.8
	**Energy AID (%)**	**83.6**	N/A	83.6

Table S5. Studies identified (n=7) comparing apparent total tract digestibility (ATTD) of macronutrients and energy in diets formulated with specific vegan (including fermented animal-based) ingredients versus animal-based diets for dogs or cats.

Note: DM=dry matter, OM=organic matter, CP=crude protein. Digestibility data are highlighted in green for diets formulated with specific vegan ingredients and red for animal-based diets. Urrego *et al.* [46] assessed a diet containing an equal mix of poultry meal and wheat gluten meal, which has been classified as animal-based in this table.

	**Urrego *et al.* (2017) [46]**	**Cargo-Froom *et al.* (2019)** [49]	**Reilly *et al.* (2021a)** [55]	**Reilly *et al.* (2021b)** [56]	**Sieja *et al.* (2023)** [62]	**Morris *et al.* (2023)** [59]	**French *et al.* (2025)** [65]
**Diet(s) formulated with specific vegan ingredients**	Extruded diet containing 13% protein from wheat gluten meal (WGM)	Extruded diet based on vegetable protein, including corn gluten meal and peas	Extruded diets containing either chickpeas (C), peanut flour (PF), green lentils (GL) or dried yeast (Y) as the main protein source	Extruded diets containing chickpea inclusion levels of 7.5% (R7.5), 15% (R15), or 30% raw chickpeas (R30), or 30% cooked chickpeas (C30)	Extruded diets containing WGM or corn gluten meal (CGM) as the protein source	Extruded diets containing rice protein concentrate (RPC) inclusion levels of 7% (R7),14% (R14) or 28% (R28)	Extruded diets containing brewed lamb protein produced via precision fermentation, at inclusion levels of 15% (L15), 30% (L30) or 40% (L40)

**Animal-based diet(s)**			Extruded diets containing 13% protein from PM, an equal mix of PM and WGM (PM+WGM), or an equal mix of PM, WGM and liver hydrolyzed protein (PM+WGM+LHP)	Extruded diet based on animal protein, including fresh beef, chicken, fish blend, whole egg and herring meal	Extruded diet containing poultry byproduct meal (PBPM) as the main protein source	Extruded diet based on PBPM and rice (0% chickpeas)	Extruded diets containing deboned, dried, and spray-dried chicken (DC) or PBPM as the protein source	Extruded diet containing hydrolyzed chicken liver and heart (0% RPC)	Extruded diet containing egg protein (0% brewed lamb protein)
**Study population and design**			8 laboratory-housed French Bulldogs (replicated 4x4 Latin square design)	8 laboratory-housed beagles (replicated 4×4 Latin square design)	10 laboratory-housed beagles (replicated 5×5 Latin square design)	10 laboratory-housed domestic shorthair cats (replicated 5x5 Latin square design)	12 laboratory-housed beagles (replicated 4×4 Latin square design)	24 laboratory-housed domestic shorthair cats (replicated 4×4 Latin square design)	6 laboratory-housed dogs per treatment
**Overall ranges**			**Individual study ranges**						
**Diet(s) formulated with specific vegan ingredients**	**DM ATTD (%)**	**77.3-90.7**	87.4	90.7	83.7 (C); 82.1 (GL); 85.6 (PF); 80.1 (Y)	83.7 (R7.5); 81.5 (R15); 81.4 (R30); 77.3 (C30)	85.4 (WGM); 84.6 (CGM)	82.7 (R7); 84.3 (R14); 85.1 (R28)	80.7 (L15); 83.2 (L30); 85.0 (L40)
	**OM ATTD (%)**	**81.7-92.2**	89.6	92.2	87.5 (C); 85.8 (GL); 90.3 (PF); 84.3 (Y)	88.6 (R7.5); 86.0 (R15); 86.4 (R30); 81.7 (C30)	90.0 (WGM); 88.3 (CGM)	N/A	N/A

	**CP ATTD (%)**	**81.5-93.8**	88.1	92.2	83.2 (C); 81.5 (GL); 85.0 (PF); 83.7 (Y)	86.2 (R7.5); 82.3 (R15); 84.5 (R30); 81.6 (C30)	93.8 (WGM); 90.1 (CGM)	N/A	87.1 (L15); 88.0 (L30); 86.7 (L40)
	**Fat ATTD (%)**	**81.0-98.0**	96.5	98	94.2 (C); 94.1(GL); 95.5 (PF); 87.9 (Y)	93.3 (R7.5); 92.8 (R15); 92.4 (R30); 91.5 (C30)	96.0 (WGM); 95.9 (CGM)	91.0 (R7); 91.7 (R14); 89.8 (R28)	85.9 (L15); 84.0 (L30); 81.0 (L40)
	**NFE ATTD (%)**	**89.0-92.8**	92.1	N/A	N/A	N/A	N/A	89.0 (R7); 90.5 (R14); 92.8 (R28)	N/A
	**Energy ATTD (%)**	**86.0-91.0**	89.6	N/A	N/A	N/A	91.0 (WGM); 89.5 (CGM)	86.0 (R7); 87.0 (R14); 87.8 (R28)	N/A
**Animal-based diet(s)**	**DM ATTD (%)**	**79.3-88.8**	85.2 (PM); 88.8 (PM+WGM); 86.2 (PM+WGM+LHP)	86.9	86.3	82.8	85.7 (DC); 81.6 (PBPM)	80.1	79.3
	**OM ATTD (%)**	**85.8-91.2**	88.1 (PM); 90.4 (PM+WGM); 88.3 (PM+WGM+LHP)	89.2	91.2	87.6	89.7 (DC); 85.8 (PBPM)	N/A	N/A
	**CP ATTD (%)**	**82.6-89.9**	84.0 (PM); 88.5 (PM+WGM); 83.6 (PM+WGM+LHP)	88.3	86.6	83.9	89.9 (DC); 82.6 (PBPM)	N/A	88.5
	**Fat ATTD (%)**	**91.0-97.1**	96.8 (PM); 96.9 (PM+WGM); 96.6 (PM+WGM+LHP)	97.1	94.7	93.6	96.6 (DC); 96.1 (PBPM)	91	92.4

	**NFE ATTD (%)**	**83.5-93.3**	91.7 (PM); 93.3 (PM+WGM); 91.8 (PM+WGM+LHP)	N/A	N/A	N/A	N/A	83.5	N/A
	**Energy ATTD (%)**	**83.6-90.6**	88.3 (PM); 90.2 (PM+WGM); 89.7 (PM+WGM+LHP)	N/A	N/A	N/A	90.6 (DC); 86.8 (PBPM)	83.6	N/A
